# Carrier Transport and Recombination Mechanism in Blue Phosphorescent Organic Light-Emitting Diode with Hosts Consisting of Cabazole- and Triazole-Moiety

**DOI:** 10.1038/s41598-019-40068-w

**Published:** 2019-03-06

**Authors:** Tian-You Cheng, Jiun-Haw Lee, Chia-Hsun Chen, Po-Hsun Chen, Po-Sheng Wang, Chuan-En Lin, Bo-Yen Lin, Yi-Hsin Lan, Yu-Hsuan Hsieh, Jau-Jiun Huang, Hsiu-Feng Lu, Ito Chao, Man-kit Leung, Tien-Lung Chiu, Chi-Feng Lin

**Affiliations:** 1 Department of Electrical Engineering, Yuan Ze University, Taoyuan, 32003 Taiwan; 2 Graduate Institute of Photonics and Optoelectronics and Department of Electrical Engineering, National Taiwan University, Taipei, 10617 Taiwan; 3 Department of Chemistry, National Taiwan University, Taipei, 10617 Taiwan; 4 Institute of Chemistry, Academia Sinica, Taipei, 11529 Taiwan; 5 Department of Electro-Optical Engineering, National United University, Miaoli, 36003 Taiwan

## Abstract

In this study, we demonstrated a blue phosphorescent organic light-emitting diode (BPOLED) based on a host with two carbazole and one trizole (2CbzTAZ) moiety, 9,9′-(2-(4,5-diphenyl-4H-1,2,4-triazol-3-yl)-1,3-phenylene)bis(9H-carbazole), that exhibits bipolar transport characteristics. Compared with the devices with a carbazole host (N,N’-dicarbazolyl-3,5-benzene, (mCP)), triazole host (3-(biphenyl-4-yl)-5-(4-tert-butylphenyl)-4-phenyl-4H-1,2,4-triazole, (TAZ)), or a physical mixture of mCP:TAZ, which exhibit hole, electron, and bipolar transport characteristics, respectively, the BPOLED with the bipolar 2CbzTAZ host exhibited the lowest driving voltage (6.55 V at 10 mA/cm^2^), the highest efficiencies (maximum current efficiency of 52.25 cd/A and external quantum efficiency of 23.89%), and the lowest efficiency roll-off, when doped with bis[2-(4,6-difluorophenyl)pyridinato-C2,N](picolinato)iridium(III) (FIrpic) as blue phosphor. From analyses of light leakage of the emission spectra of electroluminescence, transient electroluminescence, and partially doped OLEDs, it was found that the recombination zone was well confined inside the emitting layer and the recombination rate was most efficient in a 2CbzTAZ-based OLED. For the other cases using mCP, TAZ, and mCP:TAZ as hosts, electrons and holes transported with different routes that resulted in carrier accumulation on different organic molecules and lowered the recombination rate.

## Introduction

Organic light-emitting diodes (OLEDs) are a major display and lighting technology that consist of organic stacks between two electrodes^1–3^. When voltage is applied to the OLED, electrons and holes are injected into the device and recombine in the emitting layer (EML) to produce light. In an ideal case, all electrons should recombine with holes to form excitons for high efficiency^4^. Moreover, the emission zone should be as wide as possible for extending the operational lifetime^5^. In this regard, a bipolar EML is a good choice to be situated between the carrier and exciton blocking layers to confine the carriers and excitons inside the EML^6–10^. It is straightforward to use an organic material that exhibits bipolar transport characteristics as the host to achieve a “charge balance” condition. Moreover, by physically mixing hole-transporting and electron-transporting materials, it is possible to obtain the bipolar host that effectively reduces the driving voltage and extends the operational lifetime^11–13^. For a phosphorescent OLED, the dopant material also plays some role in carrier injection and transport^14–17^. For example, by using the gradient dopant technique, it is possible to broaden the recombination zone and effectively improve the operational lifetime^18,19^.

In this paper, we compare the carrier transport and recombination characteristics of two bipolar hosts in a blue phosphorescent OLED; this is an important technical bottleneck for OLED development^20–22^. One host is an “intrinsic” bipolar organic material, 9,9′-(2-(4,5-diphenyl-4H-1,2,4-triazol-3-yl)-1,3-phenylene)bis(9H-carbazole) (2CbzTAZ)^23^, that consists of two cabazole units and one triazole unit with hole- and electron-transporting characteristics, respectively, by chemical synthesis^20^. The other host is a physical mixture of N,N’-dicarbazolyl-3,5-benzene (mCP) and 3-(Biphenyl-4-yl)-5-(4-tert-butylphenyl)-4-phenyl-4H-1,2,4-triazole (TAZ) that exhibits cabazole and triazole units, respectively, fabricated by coevaporation. Additionally, single-host OLEDs with mCP and TAZ hosts were studied for comparison. Among four OLEDs fabricated with different host materials, the device made with 2CbzTAZ-EML exhibited the lowest driving voltage, highest efficiencies, and lowest efficiency roll-off. When examining the electroluminescence (EL) of the OLEDs with three other hosts (mCP, TAZ, and mCP:TAZ), emission at short wavelength (~390 nm) was observed, corresponding to the emission of the exciton/electron-blocking layer (EBL) and hole-transporting layer (HTL)^24,25^. This indicates that the carriers were leaking outside the EML without efficient recombination. In a partially doped (PD) OLED and a corresponding electron-only device (EOD), blue phosphor bis[2-(4,6-difluorophenyl)pyridinato-C2,N](picolinato) iridium(III) (FIrpic) acted in both roles—electron trap and conduction pathway—inside the mCP host, which resulted in electron-hole dislocation and reduced the efficiency of the device^26^. By observing the turned-off dynamics of transient electroluminescence (TrEL) with various reverse biases driving in the off period, the dynamics of trapped charges were investigated^27–32^. The mixed host (mCP:TAZ) effectively improved the spatial overlap of electrons and holes with less accumulated carriers than single-host OLEDs based on mCP and TAZ, trap charges inside the OLED with the 2CbzTAZ host were lowest, which explained the high efficiency and reduced efficiency roll-off. Moreover, by applying the reverse bias after the electrical pulses with different periods, it was also found that carriers may accumulate inside the device over approximately 700 µs.

## Results and Discussions

### Material characterization

**Figure 1:**
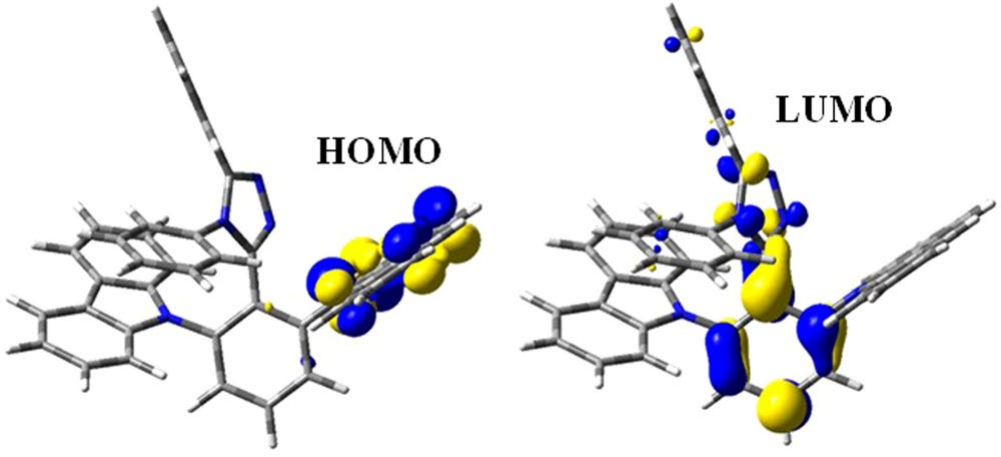
HOMO and LUMO distributions of 2CbzTAZ.

The highest occupied molecular orbital (HOMO) and lowest unoccupied molecular orbital (LUMO) distributions of the 2CbzTAZ were simulated utilizing the popular B3LYP-D3/6-31 + G* model chemistry for molecular thermochemistry, as shown in Fig. 1. The simulation plot clearly indicates that the HOMO is localized at the carbazole unit while the LUMO is located at the triazole unit.

**Figure 2:**
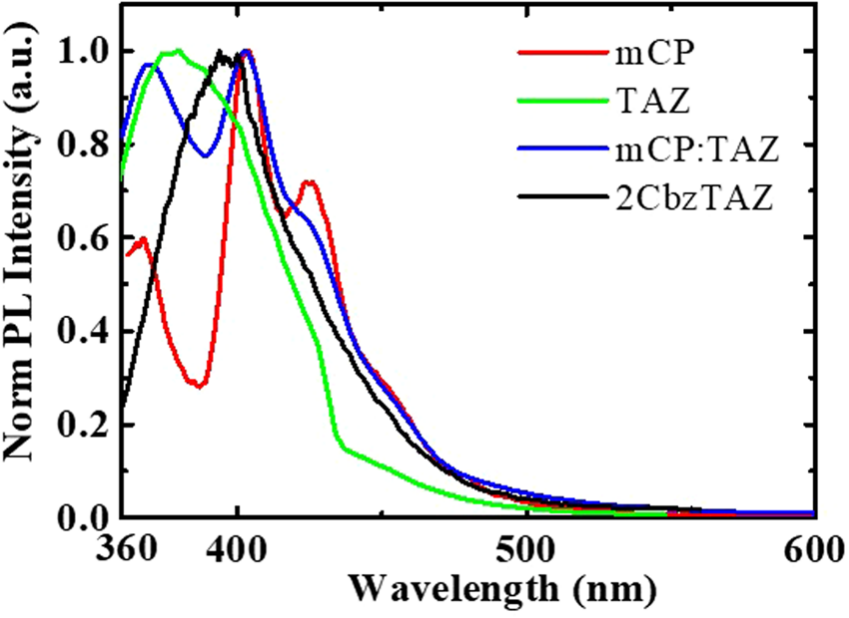
Normalized PL spectra of thin-film mCP, TAZ, mCP:TAZ, and 2CbzTAZ.

The photoluminescence (PL) spectrum of this 2CbzTAZ thin film is shown in Fig. 2. In addition, the PL spectra of three thin films (mCP, TAZ, and mCP:TAZ) were added to Fig. 2 for comparison with the 2CbzTAZ PL spectrum. The mCP:TAZ is derived from physically mixing the two carbazole and one trizole moiety together. 2CbzTAZ results from the chemical bonding of two carbazole and one trizole moiety in one molecule. The mCP PL spectral profile contains peaks at 367, 404, and 426 nm, respectively; the TAZ PL spectral profile has a single peak at 380 nm; the mCP:TAZ PL spectral profile contains peaks at 369, 403, and 423 nm, respectively; and the 2CbzTAZ PL spectral profile has a single peak at 394 nm. The physically mixed mCP:TAZ layer exhibits the PL spectral profile that combines the mCP and TAZ PL spectra. No exciplex emission can be detected. The chemical reaction in the 2CbzTAZ layer exhibits a PL spectrum as a new compound by comparing to mCP and TAZ.

### Device configurations

**Figure 3:**
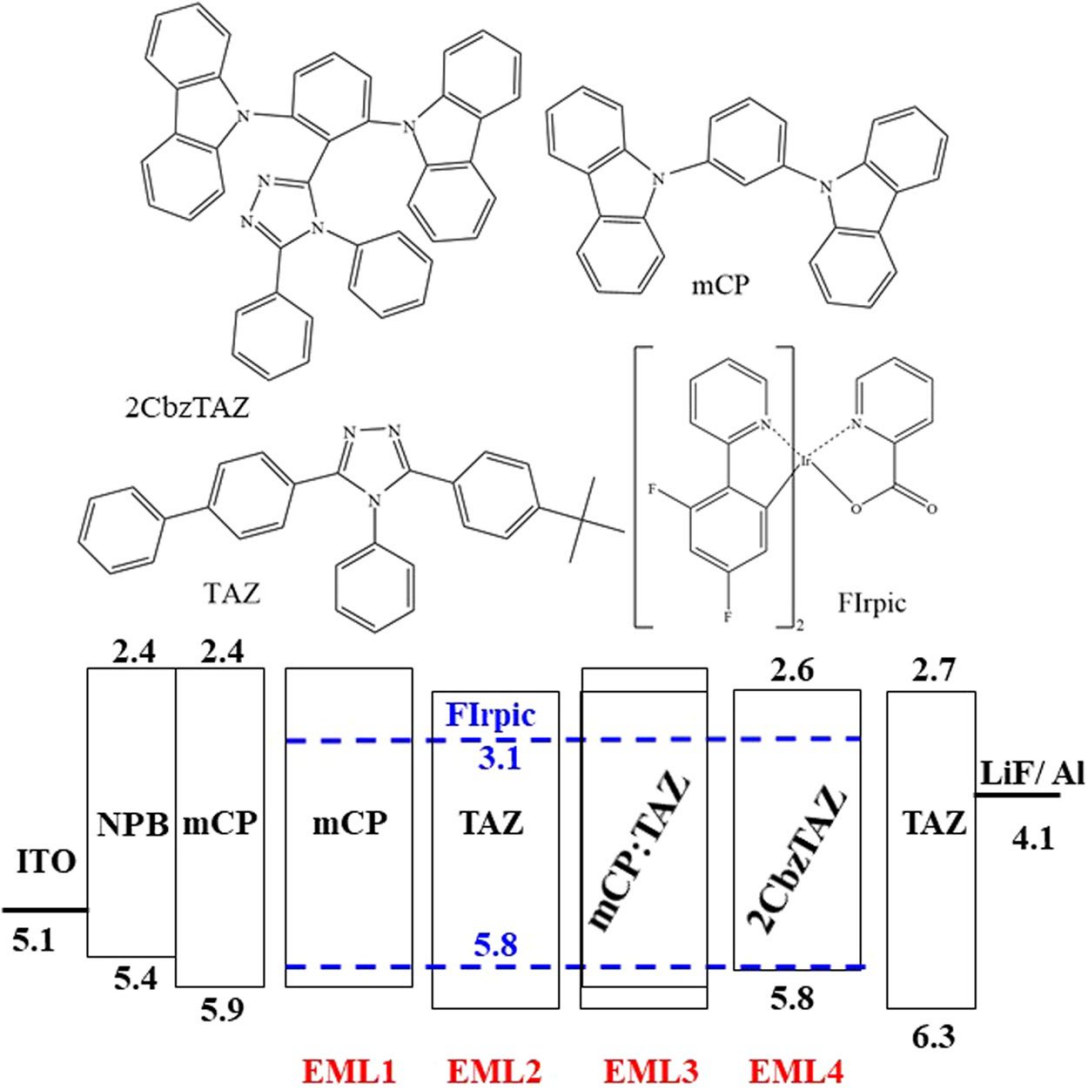
(**a**) Molecule structures and (**b**) energy levels of organic materials.

Figure 3(a,b) shows the molecular structure and energy levels, respectively. Table 1 presents the OLED structures fabricated with different host materials. Here, N,N′-diphenyl-N,N′-bis(1-napthyl)-1,1′-biphenyl-4,4′-diamine (NPB) was used as the HTL with the thickness of 50 nm. TAZ was used as the electron-transporting layer (ETL) with the thickness of 65 nm. mCP was used as the EBL between the HTL and EML with the thickness of 10 nm. Four hosts were used as the EML: mCP, TAZ, mCP:TAZ, and 2CbzTAZ, with the thickness of 35 nm and doped with 15% Firpic in volume ratio. 2CbzTAZ consisted of two cabazole units and one triazole unit connected with the central benzene ring^23^. The mixing ratio of mCP:TAZ was 1:2, which was determined from device optimization. LiF and Al with thicknesses of 0.9 and 100 nm were used as the electron injection layer (EIL) and cathode, respectively. In our experiments with the PD-EML of the mCP host, the device structures were NPB (50 nm)/mCP (10 nm)/EML (30 nm)/TAZ (30 nm). For the EOD, device structures were Al (50 nm)/LiF (0.9 nm)/mCP (10 nm)/EML (30 nm)/TAZ (30 nm)/LiF (0. 9 nm)/Al (120 nm). In these two cases, EML was divided into three regions, each of 10 nm, and selectively doped (denoted as “D”) or undoped (denoted as “U”) with 15% FIrpic molecules into the mCP matrix.

**Table 1:** Layer structures of the devices.

BPOLED	NPB		mCP	Host: FIrpic 35 nm 15%			TAZ	LiF	Al
mCP	50 nm		10 nm	mCP			65 nm	0.9 nm	120 nm
TAZ				TAZ					
mCP:TAZ				mCP:TAZ					
2CbzTAZ				2CbzTAZ					
**PD-EOD**	**Al**	**LiF**	**mCP**	**mCP: X% FIrpic**			**TAZ**	**LiF**	**Al**
				**10** **nm**	**10** **nm**	**10** **nm**			
UUU	50 nm	0.9 nm	10 nm	0%	0%	0%	30 nm	0.9 nm	120 nm
DDD				15%	15%	15%			
UUD				0%	0%	15%			
DUD				15%	0%	15%			
**PD-OLED**	**NPB**		**mCP**	**mCP: X% FIrpic**			**TAZ**	**LiF**	**Al**
				**10** **nm**	**10** **nm**	**10** **nm**			
UUD	50 nm		10 nm	0%	0%	15%	30 nm	0.9 nm	120 nm
**UDU**				**0%**	**15%**	**0%**			
**DUU**				**15%**	**0%**	**0%**			
**DDD**				**15%**	**15%**	**15%**			

### Electrical and optical performance of BPOLEDs with different hosts

**Figure 4:**
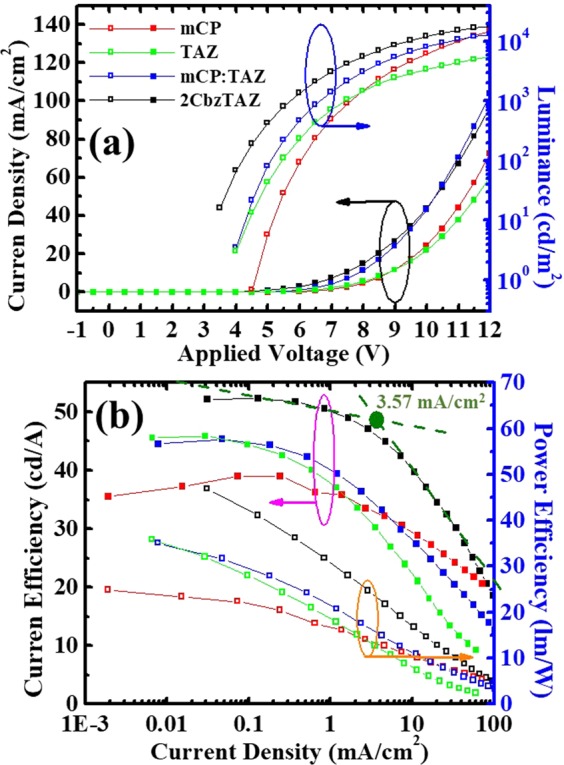
(**a**) Current density and luminance versus voltage, and (**b**) current efficiency (cd/A) versus luminance of BPOLEDs with different hosts.

Figure 4(a) shows the L-J-V characteristics of BPOLEDs with different hosts. Corresponding device performances are summarized in Table 2. Two groups of J-V characteristics are presented in Fig. 4(a). For the mCP- and TAZ-hosts with unipolar carrier transport, driving voltage was higher (8.05 V and 7.87 V at 10 mA/cm^2^, respectively). With the introduction of Cbz and TAZ moieties together as the host of the EML, physically mixed (mCP:TAZ) or chemically synthesized (2CbzTAZ), the driving voltage of the BPOLEDs effectively decreased to 6.99 V and 6.55 V at 10 mA/cm^2^, respectively, because of the bipolar carrier transport characteristics. For the L-V curves in Fig. 4(a), luminance was always highest for the 2CbzTAZ case, which implied high efficiency. Comparing the BPOLED with mCP and TAZ hosts, the TAZ host exhibited higher and lower luminance under low and high driving voltages, respectively, because of the narrower recombination zone, which is discussed later. Figure 4(b) shows current and power efficiency (in terms of cd/A and lm/W, respectively) versus current density for the four BPOLEDs. The maximum current efficiency of the blue BPOLED with mCP host was 39.02 cd/A at luminance of 94.12 cd/m^2^, which was comparable to other studies that used similar material systems and device structures^33^. When replacing the host to TAZ, the maximum efficiency increased to 45.90 cd/A at luminance of 13.44 cd/m^2^. Although the maximum current density was higher in the TAZ-OLED compared with the mCP-OLED, the efficiency roll-off was more serious in the TAZ-based device. mCP is basically a hole-transporting material that also exhibits a certain electron transport capability that broadens the recombination zone^34^. We also needed to consider the effects of FIrpic in the matrix for carrier transport, which is discussed in the next section. TAZ is a pure electron-transporting material that blocks the hole, and when it was used as the host of the EML, holes injected through FIrpic molecules had low hole mobility and thus the recombination zone was very narrow. However, such trap-assisted recombination was effective for carrier confinement, which can achieve high efficiency under a low driving condition. When mCP and TAZ were physically mixed together, current efficiency was similar to the case of TAZ under low luminance (<100 cd/m^2^). Under high current density driving (~2000 cd/m^2^), current efficiency was between the OLEDs with mCP- and TAZ-hosts, which can be also shown from the efficiency roll-off (between maximum current efficiency and that at 1000 cd/m^2^), as indicated in Table 2. The device with 2CbzTAZ host showed higher current efficiency, under both low and high current density, and alleviated efficiency roll-off, which meant the emission mechanism for this case was different from the others. In particular, this 2CbzTAZ device exhibited superior device performance (e.g., maximum current efficiency and external quantum efficiency of 52.25 cd/A and 23.89%, respectively) to that of the device in our previous report^23^ (maximum current efficiency and external quantum efficiency of 43.3 cd/A and 17.9%, respectively). Furthermore, the advanced physical insights of devices were investigated using steady EL spectrum and transient EL signals under various biases in later discussion.

**Table 2:** Device performances of the BPOLEDs with different hosts.

	Driving Voltage (V)	Current Efficiency (cd/A)	EQE (%)^d^	Roll-off (%)^e^
mCP	6.11^a^, 7.72^b^, 8.05^c^	39.02^d^, 38.11^a^, 32.82^b^	18.03	15.89
TAZ	5.31^a^, 7.23^b^, 7.87^c^	45.90^d^, 43.36^a^, 31.77^b^	20.22	30.78
mCP:TAZ	5.24^a^, 6.72^b^, 6.99^c^	46.10^d^, 43.21^a^, 34.64^b^	20.29	24.86
2CbzTAZ	4.23^a^, 5.74^b^, 6.55^c^	52.25^d^, 51.97^a^, 47.96^b^	23.89	8.21

^a^Luminance at 100 cd/m^2^, ^b^Luminance at 1000 cd/m^2^, ^c^Measured at 10 mA/cm^2^, ^d^Maximum value, ^e^(CE_Max_ − CE_1000_)/CE_Max_, CE_Max_ and CE_1000_: current efficiency at maximum value and 1000 cd/m^2^, respectively.

**Figure 5:**
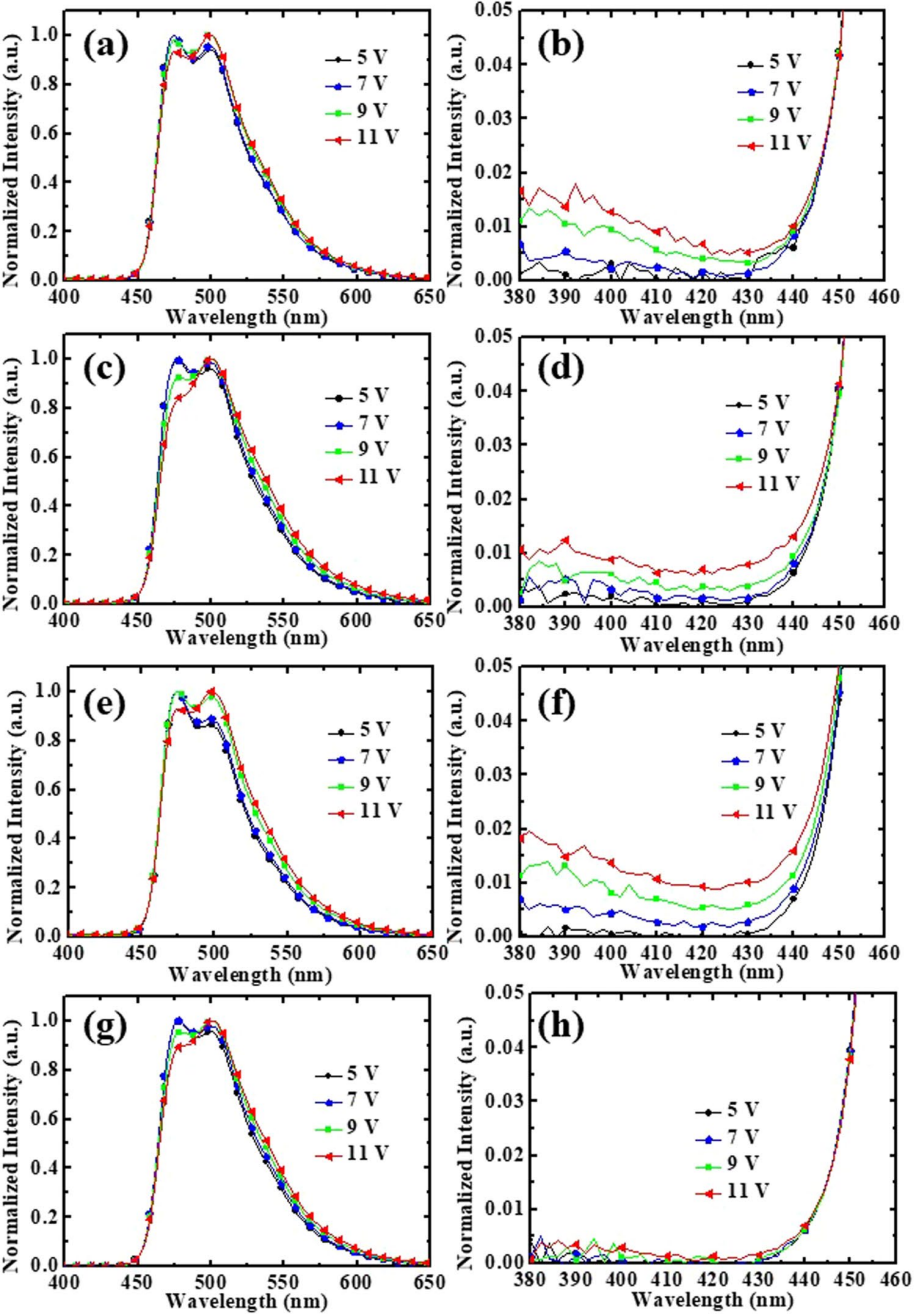
EL spectra at different driving voltages for BPOLEDs with different hosts: (**a**) mCP, (**c**) TAZ, (**e**) mCP:TAZ, and (**g**) 2CbzTAZ. (**b**), (**d**), (**f**), and (**h**) are zoomed-in spectra at short wavelength region (380–460 nm) of (**a**), (**c**), (**e**), and (**g**), respectively.

Figure 5a,c,e,g showed the EL spectra of the OLEDs with mCP-, TAZ-, mCP:TAZ-, and 2CbzTAZ-hosts with different voltage driving conditions. There were two emission peaks at 476 and 500 nm for FIrpic emission. Figure 5b,d,f,h showed the zoomed-in EL spectra in the short wavelength region and that emission of EBL (pure mCP with a peak of ~380 nm) and even HTL (NPB with a peak of ~420 nm) were observed with higher driving voltage^24^. When using TAZ as the host of the EML (Fig. 5d), it was an intrinsically ETL material. Holes were injected into the EML through FIrpic molecules and hence the major recombination zone was close to the HTL/EML interface. It was reasonable to observe mCP and NPB emissions in this case, as shown in Fig. 5d, because some (minor) recombination happened inside the EBL (mCP) and even HTL (NPB). For the mCP case (Fig. 5b) that exhibited higher hole mobility than electron mobility, the main recombination zone took place in the EML close the ETL side. Light leakage was still observable in Fig. 5b, because electrons transported through the FIrpic molecules (this is discussed in the next section), and thus did not completely recombine with holes (transporting mainly on the mCP) in the EML. When mixing mCP and TAZ as the co-host EML, such leakage was still observed in Fig. 5e, because the recombination zone shifted toward the anode side compared to the mCP-case with introducing the electron-transporting TAZ. However, when using 2CbzTAZ as the bipolar host, the intensity of the leakage emission was greatly suppressed (0.5% compared with the main FIrpic emission), as shown in Fig. 5h, meaning that the carrier transport and recombination processes were quite different for mCP:TAZ and 2CbzTAZ, although both hosts exhibited bipolar transport characteristics. For mCP:TAZ, holes and electrons transported among different molecules reduced the recombination efficiency and hence light leakage was observed. For 2CbzTAZ, both holes and electrons transported on the 2CbzTAZ, which facilitated carrier recombination without leaking outside the EML. In particular, it could be detected that the variation of the EL peak form 476 to about 500 nm in Fig. 5a,c,e,g, when the driving voltage was increased. According to the charge carrier mobility in the organic thin film, the mobility of electrons and holes depends on the electrical field^35^. When the electrical field increases, the mobility of electrons and holes increases. Generally, electron mobility increases faster than hole mobility in organic thin films. For 2CbzTAZ, electron mobility also exhibited a more increased slope than that of holes^23^. Therefore, with increasing voltage, the recombination zone in the EML shifts toward the anode side and the emission spectrum becomes redshift because of the microcavity effect inside the device^24,36^.

**Figure 6:**
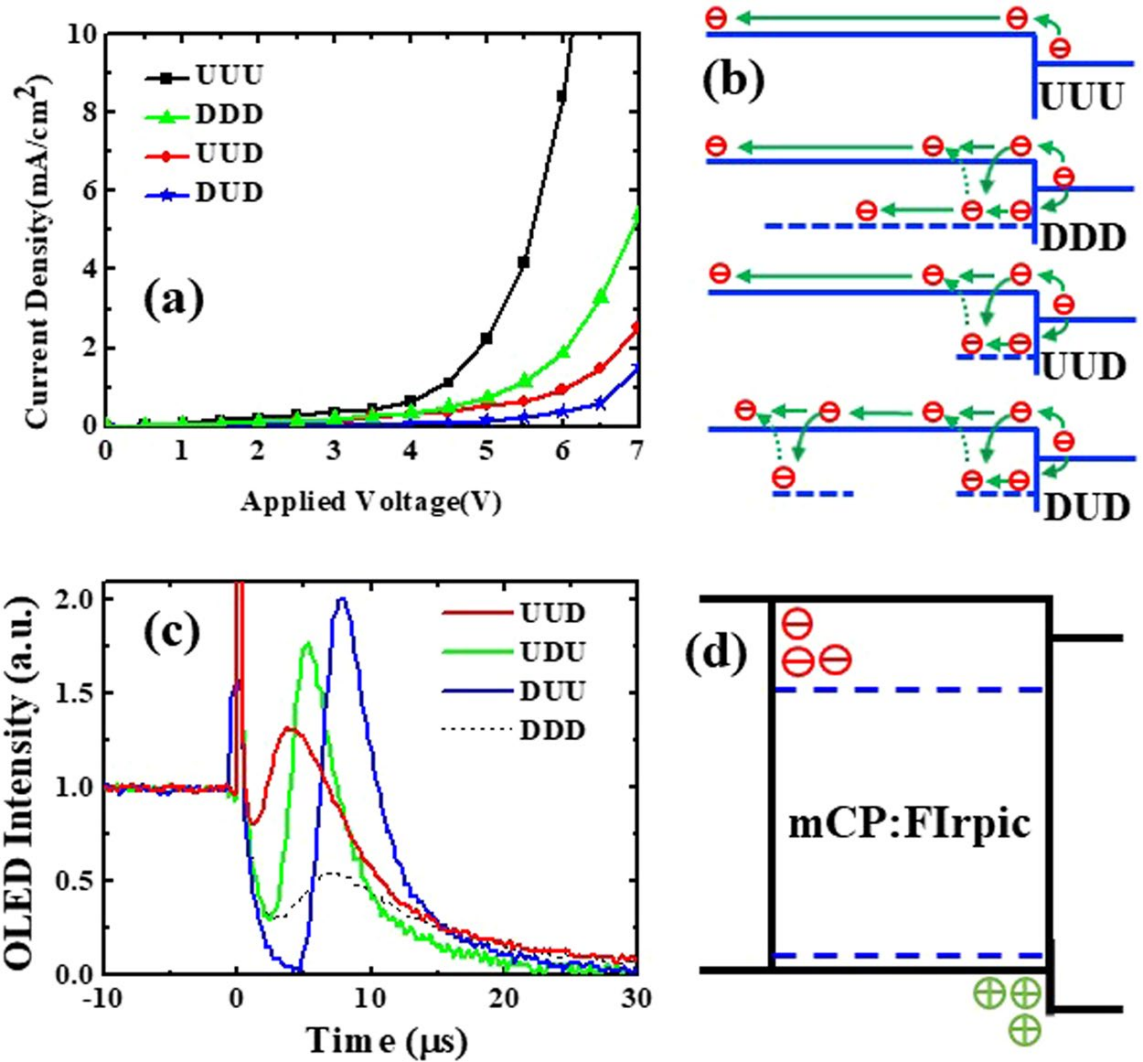
(**a**) J-V characteristics of EODs. (**b**) Schematic diagram of electron transport in different EODs. (**c**) Transient luminance of different PD-OLEDs when the electrical pulse was turned off. (**d**) Schematic diagram of electron and hole distribution in a BPOLED with mCP:FIrpic EML.

To further understand the dopant effect for electron transport in blue phosphorescent OLED, we fabricated the mCP-based EODs with FIrpic selectively doped inside the EML^26^. “U” and “D” meant the undoped and doped region from the anode to cathode side. As shown in Fig. 6a, compared with the uniformly doped (denoted as “DDD”) and undoped (denoted as “UUU”) OLEDs, driving voltage increased with the incorporation of FIrpic, which showed the characteristics as carrier trapping sites. However, when selectively doped FIrpic was close to the cathode side (UUD case), the driving voltage was higher than that in the case of a uniformly doped OLED (DDD case), which meant that FIrpic also played the role in electron transport^37^. Hence, we proposed the electron transport model for the FIrpic-doped mCP layer, as shown in Fig. 6b. For the undoped case, electrons hopped on the mCP site with certain electron mobility. When incorporating FIrpic in mCP uniformly, electrons were trapped by Firpic because of an energetically favorable process and transported through the FIrpic sites, which meant electrons transported at two channels: mCP and FIpric. When selectively doping Firpic in the EML close to the ETL interface, electrons were trapped by FIrpic that could not transport further and it was difficult to detrap to the mCP site. Thus, it acted as the space charge which impeded the subsequent electrons and increased the driving voltage (UUD case). Furthermore, we doped both sides and left the center part undoped (DUD case). Then, the driving voltage was highest because there were two separate trapping regions that impeded the conduction paths of electrons among FIrpic sites. Figure 6c shows the EL intensity during the turned-off transient of the mCP-based OLED that was selectively doped with FIrpic at different regions. After the voltage was turned off at 0 µs, EL intensity initially decreased, which was followed by a peak at 5–10 µs attributable to the recombination of trapped carriers^28^. The doped region was filled with electrons under forward bias (at t < 0 µs) that escaped the traps after turning off the forward bias and recombined with the holes accumulated at the EML/ETL interface. Thus, with the doping region away from the cathode side from UUD, UDU, to DUU, the “peak” time increased from 3.7, 5.3, to 7.6 µs due to longer distances for electron and hole diffusion. Moreover, the peak intensity relative to the steady-state intensity (at t < 0 µs) increased when the doped region shifted to the anode side. Because the main recombination zone was close to the EML/ETL interface, steady-state intensity was highest for the UUD case, which resulted in a lower relative intensity of the transient peaks. For the uniformly doped OLED (DDD case), the efficiency was highest and thus the relative peak intensity was lowest. Additionally, the peak was broad because of the broad doped region. In such a device, electrons and holes mainly transported on FIrpic and mCP molecules, respectively, which results in poor spatial overlap of electron and holes, as shown in Fig. 6d, and hence lower recombination and efficiency.

**Figure 7:**
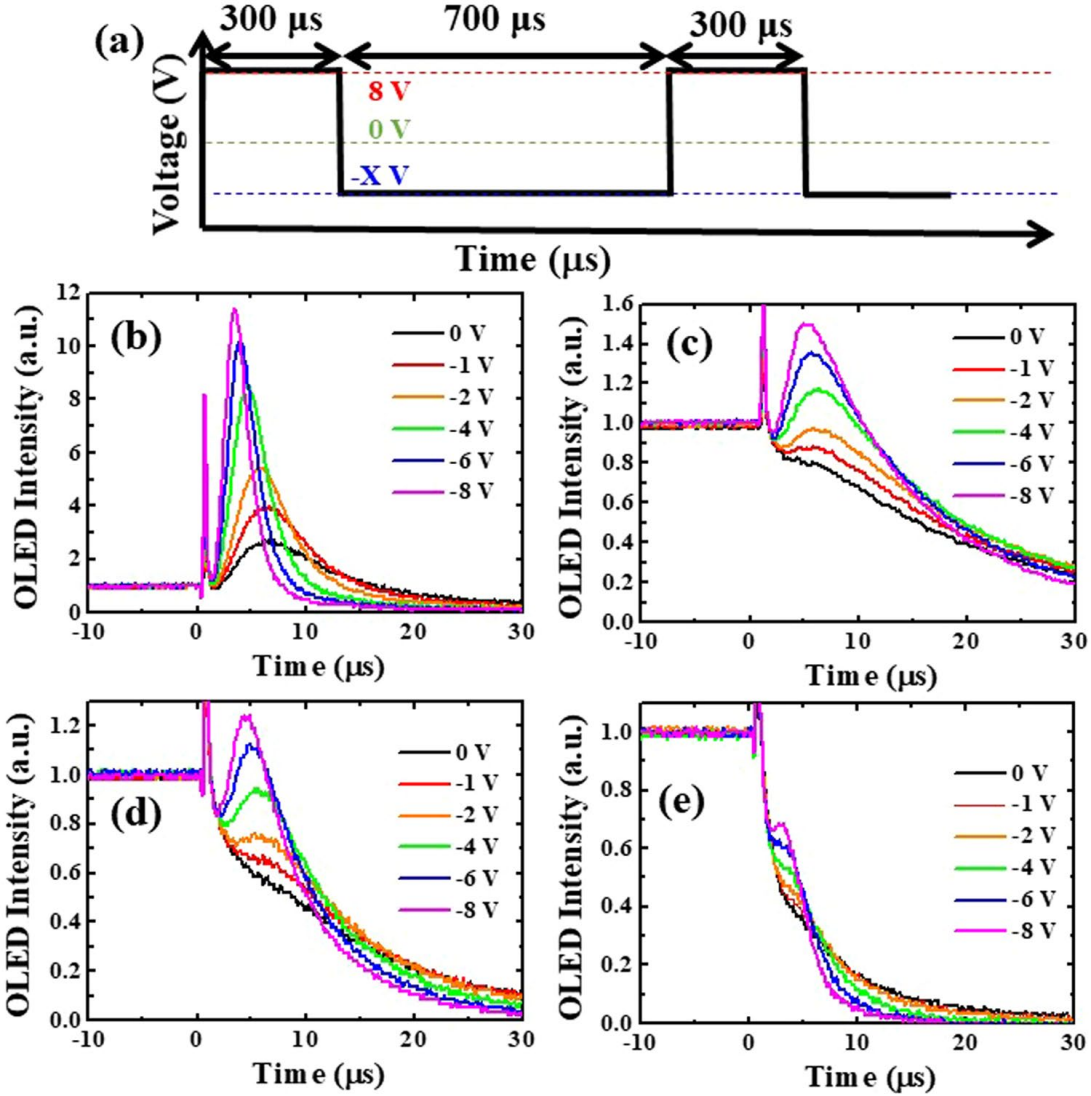
(**a**) Schematic diagram of waveforms with different reverse biases during off period. Transient luminance of BPOLEDs with (**b**) mCP, (**c**) TAZ, (**d**) mCP:TAZ, and (**e**) 2CbzTAZ when the electrical pulse was turned off under different driving waveforms.

We investigated the turned-off dynamics of TrEL for BPOLEDs with different hosts under different driving waveforms, as shown in Fig. 7a. The turn-on and -off durations were 300 and 700 µs, respectively, which corresponded to a 1 kHz repetition rate and 30% duty cycle. BPOLEDs were biased at 8 V during the on period, and then were applied on various reverse biases (0 to −8 V) starting at t = 0 µs. Two spikes were detected. The first sharp spike signal near t = 0 µs was induced by electromagnetic signal coupling, happened in a short time, and did not belong to the carrier dynamics; thus, it can be ignored. The second Gaussian spike signal was deduced by the detrapped charge carrier dynamics and recombination because the trapped carriers in the EML or layer interfaces were extracted out under a reverse bias^28^. Therefore, the second spike can be understood as the result of trapped carriers. The intensity of the transient EL signal represents how many trapped carriers were inside the device. During the off period, a reverse bias was applied to the device. It accelerated the carrier transport of detrapped charges, which resulted in a stronger peak intensity and shorter time to achieve the intensity peak, as shown in Fig. 7b–e. For the mCP-OLED with −8 V bias in the off period, the peak intensity reached 11 times that of the steady-state because of poor electron and hole overlap in Fig. 7b. For the TAZ-OLED, the peak intensity was lower because the recombination was narrower, which also echoed the high efficiency at low current density and serious efficiency roll-off at high current density. For the OLED with a mixed host, the peak intensity was lower than the TAZ-based device but still higher than the steady-state intensity under −8 V reverse bias. For the mCP:TAZ-OLED, holes mainly transported through the mCP molecules with FIrpic as the shallow hole trap because of a small difference in HOMO levels (0.1 eV). TAZ molecules have negligible hole mobility and serve as the “blocking hills” to the holes. For the electrons, TAZ molecules exhibited much higher electron mobility than mCP and FIrpic. At the same time, FIrpic molecules were the deep electron traps for the mCP:TAZ matrix. Compared with the pure mCP OLED, holes were retarded by TAZ molecules for better carrier recombination in the mCP:TAZ device. Compared with the pure TAZ one, incorporation of mCP molecules in the mixed host structure improved hole-concentration into the EML which broadened the recombination zone and hence alleviated the efficiency roll-off.

An unsolved problem for the physically mixed host structure was the leakage recombination outside the EML due to complex carrier transport (especially for the electron) characteristics in such three-molecules (mCP, TAZ, FIrpic) thin-film. No exciplex formation between mCP and TAZ, as the PL results in Fig. 2, meaning that recombination from hole and electron on mCP and TAZ, respectively, was not possible^38,39^. A similar situation happened between mCP and FIrpic, as well as Firpic and TAZ. This meant that we considered only the electron and hole transport characteristics (carrier concentration) on different molecules at different positions of the EML. In another word, electron-hole recombination happened on the same molecules. Chemically synthesized cabazole and triazole units combined as a 2CbzTAZ molecule exhibited unique HOMO and LUMO values, and it is evident that the turned-off spike was greatly suppressed, as shown in Fig. 7e, compared with the other three cases (mCP, TAZ, and mCP:TAZ in Fig. 7b–d). Two major differences were apparent between BPOLEDs using physically mixed mCP:TAZ and chemically synthesized 2CbzTAZ as hosts. The first was the carrier transport characteristics. In the 2CbzTAZ system, holes transported through the host because of the high hole mobility with the same HOMO value as the FIrpic. On the other hand, electrons transported on the host too, but partially trapped by FIrpic molecules. Those were much simpler than the case in the mixed-host OLED with three molecules. The second difference was related to recombination because it happened in one molecule (2CbzTAZ), which alleviated the carrier accumulation on different molecules (mCP and TAZ in the mCP:TAZ host). Hence, due to simpler carrier transport channels and enhanced recombination efficiency with the 2CbzTAZ host, which can be deduced from TrEL intensity and the EL spectra, BPOLED with higher efficiency can be achieved.

**Figure 8:**
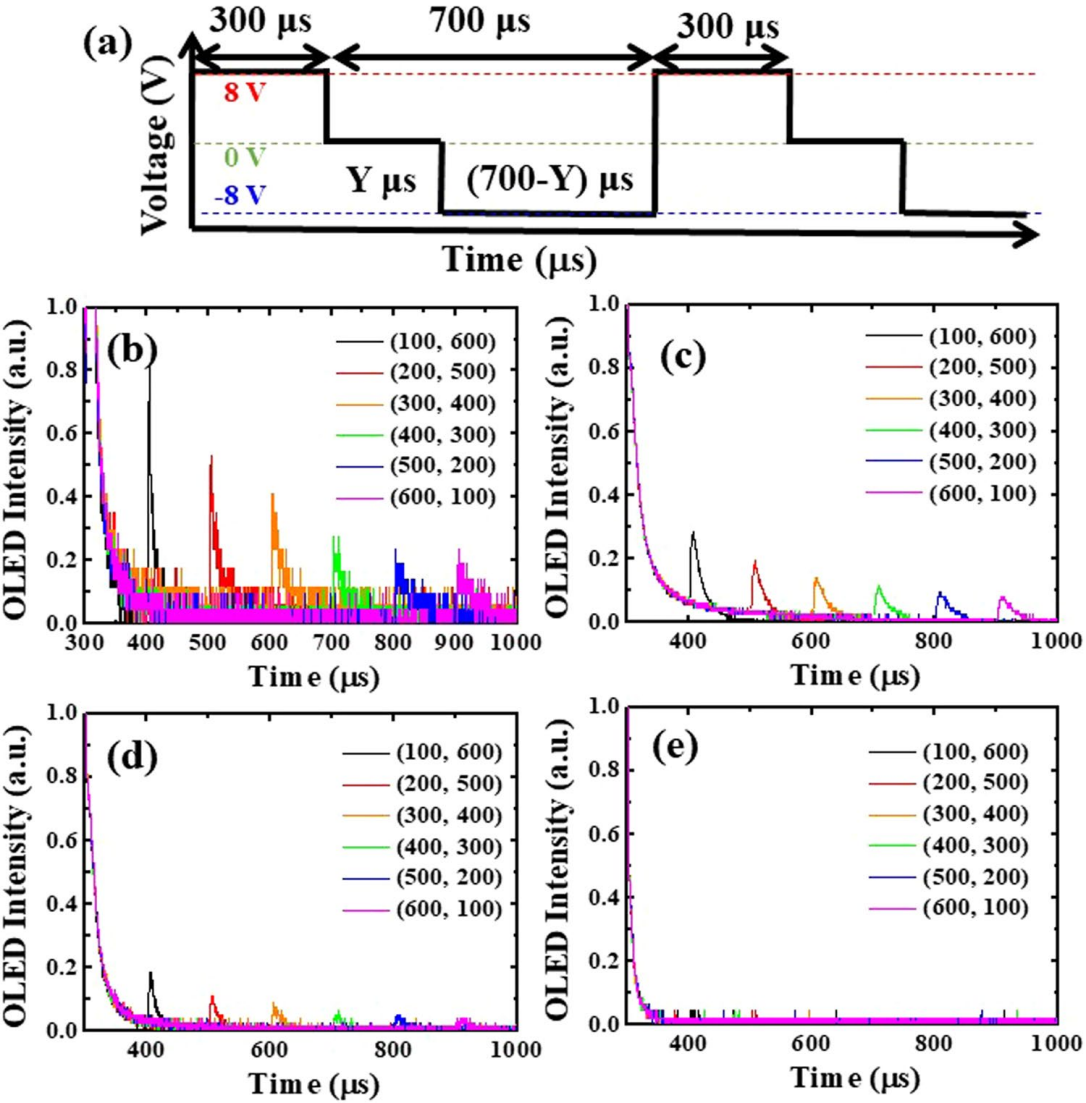
(**a**) Schematic diagram of waveforms with different times for 0 V and −8 V during the off period. Transient luminance of BPOLEDs with (**b**) mCP, (**c**) TAZ, (**d**) mCP:TAZ, and (**e**) 2CbzTAZ when the electrical pulse was turned off under different driving waveforms.

We used different driving waveforms to investigate how long those trapped carriers stored in the BPOLEDs, as shown in Fig. 8a. After the device was turned off, it was held at 0 V for a certain time (100 to 600 µs), which was much longer than the relaxation time of the trap-induced recombination time (~30 µs) in Fig. 8b–e. Then, a reverse bias (−8 V) was applied to sweep out the remaining trapped charges. For the mCP case, the spikes were strongest, which was consistent with the results in Fig. 8. Furthermore, the spikes decreased when using TAZ and mCP:TAZ. The spike intensity decreased when increasing the 0 V duration from 100 to 600 µs, which implied the exciton relaxation was nonradiatively and also explained the lower efficiency of these three cases. Before the next forward bias was applied, some remaining charges were in the OLED, which also affected the electrical characteristics of such a device^40^. On the other hand, by using 2CbzTAZ, no spike was observed, which meant there was no carrier accumulation in this device, indicating the high efficiency of the BPOLED with this host.

### Summary

In summary, we investigated the carrier transport and recombination process in the blue phosphorescent material with different hosts exhibiting hole (mCP), electron (TAZ), and bipolar (mCP:TAZ and 2CbzTAZ) transport characteristics. For bipolar hosts, it may consist of co-evaporation of hole- and electron-transport materials (mCP:TAZ) or the chemical connection of hole- and electron-transport moiety (2CbzTAZ). The dopant (FIrpic) effect had to be considered in such a device for carrier transport, which resulted in carrier accumulation in different molecules for the cases of mCP, TAZ, and mCP:TAZ and reduced the recombination rate and efficiency. It was analyzed from the following perspectives: (1) light leakage at short wavelength range (380–450 nm) of EL spectra, (2) the EOD and turned-off dynamics of the TrEL of PD devices, and (3) turned-off dynamics of BPOLEDs. Carrier accumulation was alleviated for BPOLEDs that used 2CbzTAZ as the host, achieving the maximum current efficiency of 52.25 cd/A and EQE of 23.89%.

## Method

### 2CbzTAZ Synthesis

**Figure 9:**
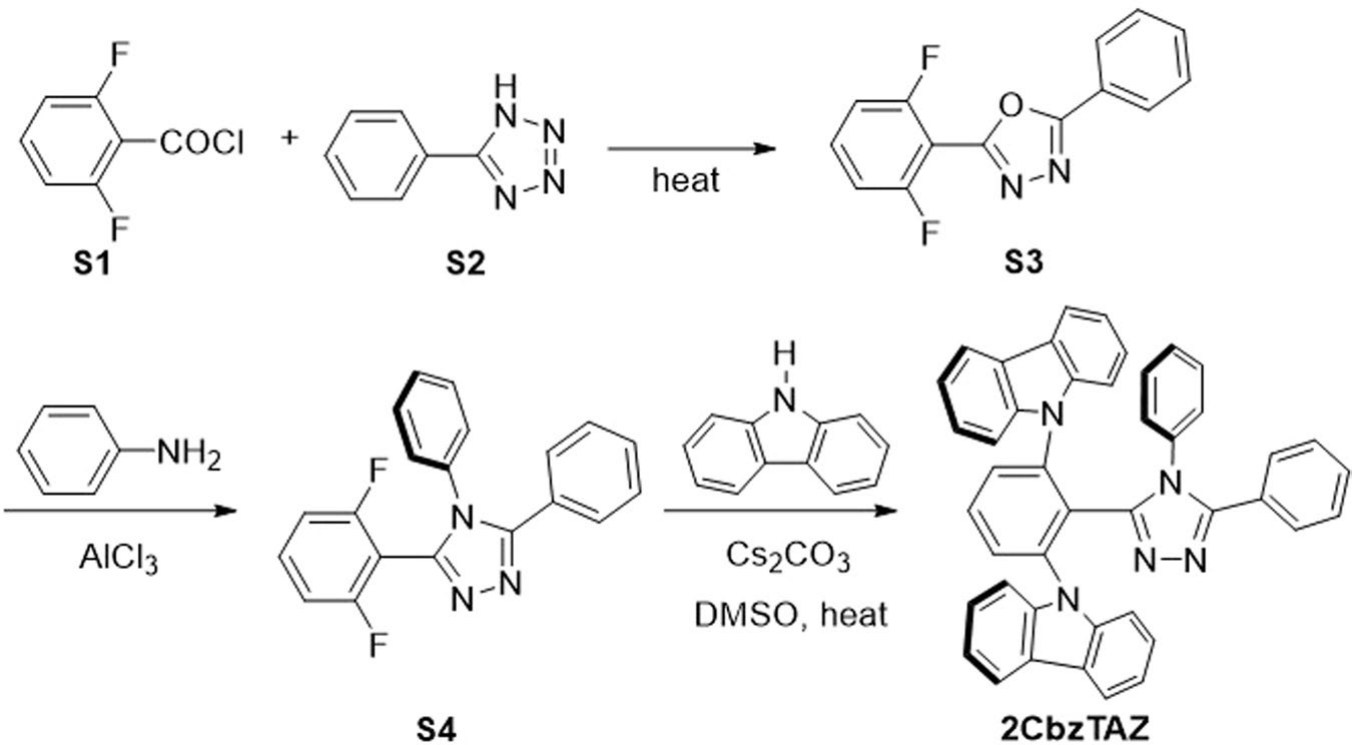
Synthesis of 2CbzTAZ.

The synthesis procedure of 2CbzTAZ as shown in Fig. 9. To a solution of S1 (4.55 g, 31.1 mmol) in pyridine (30 mL) was added 2,6-difluorobenzoyl chloride (S2) (3.56 mL, 28.3 mmol) under a nitrogen atmosphere. The mixture was then heated under reflux conditions (at 90 °C) for 24 h. The mixture was cooled and precipitated in diluted aqueous HCl. Sligthly yellowish solid was precipitated under these conditions. The crude solid was collected by suction filtration. Recrystallization of the solid from acetone, followed by rinsing with methanol gave the essentially pure S3 as colorless needles (5.86 g, yield 80%) mp. 117–118 °C; ^1^H NMR (400 MHz, *d*_6_-DMSO) δ 7.95–8.15 (m, 2H), 7.55–7.75 (m, 4H), 7.30–7.40 (m, 2H); ^13^C NMR (100 MHz, *d*_6_-DMSO) δ 164.36, 159.75 (dd, *J* = 192, 5.3 Hz), 156.18 (t, *J* = 4 Hz), 134.52 (t, *J* = 10 Hz), 132.10, 129.74, 126.06, 122.79, 112.75 (dd, *J* = 24.4, 3.3), 102.03 (t, *J* = 16 Hz); MS (EI) 258 (M^+^); HRMS (EI) calcd for C_14_H_8_F_2_N_2_O 258.0605 (M^+^), obsd. 258.0603. Anal. calcd for C_14_H_8_F_2_N_2_O: C, 65.12; H, 3.12; N, 10.85; found C, 65.28; H, 3.04; N, 10.79.

#### 3-(2,6-Difluorophenyl)-4,5-diphenyl-4*H*-1,2,4-triazole (S4)

A mixture of aniline (1.86 mL, 20.4 mmol) and aluminum chloride (0.68 g, 5.10 mmol) was stirred at 160 °C under argon for 2.5 h. 2-(2,6-Difluorophenyl)-5-phenyl-1,2,4-oxadiazole (S3) (2.00 g, 7.75 mmol) in *N*-methylpyrrolidinone (1.55 mL) was added to the mixture and then heated at 200 °C for 24 h. The reaction mixture was poured into ice water and the precipitated crude product was collected and dried. The crude product was washed with diluted aqueous HCl and water. The precipitate was collected by suction filtration and recrystallized from methanol to give a solid, which was further washed with a portion of acetone and dried to give essentially pure 3-(2,6-difluorophenyl)-4,5-diphenyl-4*H*-1,2,4-triazole (S4) as white crystalline (2.15 g, yield 83%): mp. 224–225 °C; ^1^H NMR (400 MHz, *d*_6_-DMSO) δ 7.59 (m, 1H), 7.46–7.35 (m, 8H), 7.26 (d, *J* = 6.84, 2H), 7.19 (t, *J* = 8.08, 2H); ^13^C NMR (100 MHz, *d*_6_-DMSO) δ 160.13 (dd, *J* = 187.5, 5.7 Hz), 154.48, 145.10, 134.10 (t, *J* = 10.0 Hz), 129.92, 129.73, 128.61, 128.36, 127.01, 126.32, 111.99 (d, *J* = 19.21), 104.85 (t, *J* = 20.0 Hz); MS (EI) 333 (M^+^); HRMS (EI) calcd for C_20_H_13_F_2_N_3_ 333.1078 (M^+^), obsd. 333.1074. Anal. calcd for C_20_H_13_F_2_N_3_: C, 72.06; H, 3.93; N, 12.61; found C, 72.06; H, 3.93; N, 12.71.

#### 1,3-Bis(9*H*-carbazolyl)-(2-(4,5-diphenyl-4*H*-1,2,4-triazol-3-yl)benzene (2CbzTAZ)

A mixture of Cs_2_CO_3_ (3.07 g, 9.43 mmol) and carbazole (1.45 g, 8.68 mmol) in DMSO (12 ml) was stirred at room temperature under argon for 30 min. S4 (1.40 g, 4.20 mmol) was added to the mixture and then heated at 160 °C for 72 h, and crystalline product was formed. The reaction mixture was poured into a mixture of ice-water and diluted HCl. The crude product collected as solid, washed with water, and dried. The product was further purified by recrystallization from CH_2_Cl_2_ and acetone to afford a solid, which was further washed with one portion of acetone and dried under reduced pressure to give white crystalline product (2.21 g, 79%). mp. 342–343 °C; ^1^H NMR (400 MHz, CD_2_Cl_2_) δ 8.12 (t, *J* = 6.96, 4H), 7.83–7.76 (m, 3H), 7.62–7.58 (m, 4H), 7.36 (dd, *J* = 7.4, 5.98, 4H), 7.29, (t, *J* = 7.36, 2H), 7.11 (t, *J* = 7.44, 1H), 7.05 (t, *J* = 7.52, 1H), 6.99–6.96 (m, 4H), 6.79 (dd, *J* = 8.88, 7.84, 4H), 6.18 (d, *J* = 7.64, 2H); ^13^C NMR (100 MHz, CD_2_Cl_2_) δ 153.92, 148.86, 142.10, 142.08, 140.68, 133.21, 133.02, 130.45, 129.41, 129.21, 129.11, 128.56, 128.01, 127.82, 126.82, 126.31, 126.19, 126.03, 124.44, 123.66, 120.67, 120.54, 120.46, 119.95, 112.50, 110.29; HRMS m/z [M + H]^+^ 628.2501 Anal. calcd for C_44_H_29_N_5_: C, 84.19; H, 4.66; N, 11.16; found C, 84.10; H, 4.65; N, 11.19.

### Device fabrication and measurement

The OLEDs were fabricated on the glass substrate with patterned indium-tin-oxide (ITO) thin film as the anode^41^. Oxygen plasma was employed on the ITO surface before the organic thin film for increasing the work function. Thin film stacks and Al cathode were thermally deposited in a multi-source vacuum chamber. Then, the samples were sent to N_2_ glove box for encapsulation process. Current density-voltage (J-V) characteristics of the OLEDs under steady state were obtained by a source meter (Keithley 2400). Luminance and spectra of the EL were measured by a spectroradiometer, Minolta CS-1000. For TrEL measurement, voltage waveforms were supplied with Agilent 33509B. Time-dependent emission from the OLEDs were obtained from the photomultiplier tube (PMT), Hamamatsu H6780–20, connected to the oscilloscope (Tektronix TDS 2004C)^42^.
